# Myocardial velocity mapping for the right ventricle in pulmonary arterial hypertension using a novel image-based respiratory self-navigation from a golden-angle spiral acquisition

**DOI:** 10.1186/1532-429X-15-S1-P43

**Published:** 2013-01-30

**Authors:** Daniel S Knight, Jennifer A Steeden, Shreya Bali, Andrew M Taylor, Vivek Muthurangu

**Affiliations:** 1 UCL Centre for Cardiovascular Imaging, University College London, London, UK; 2 Division of medicine, University College London, Royal Free Campus, London, UK

## Background

CMR is a recognized method of assessing global RV function in patients with pulmonary arterial hypertension (PAH). Unfortunately, assessment of regional myocardial motion with MRI remains challenging due to the thin RV wall. One solution may be tissue phase mapping (TPM) in which motion can be encoded at a higher spatial resolution. However, one problem is the need for respiratory navigation due to long scan times. We have therefore implemented a novel self-gated golden-angle spiral TPM technique that can acquire high-resolution data efficiently during free breathing in healthy volunteers and in patients with PAH.

## Methods

TPM was performed using a rotating golden-angle spiral acquisition. Reconstruction of real-time images allowed creation of an image-based respiratory navigator, which was used to select expiratory spiral interleaves. Expiratory data was retrospectively cardiac-gated using data binning. TPM of a mid-ventricular slice was acquired in 18 PAH patients and 10 healthy volunteers. Post-processing of TPM data was performed on in-house software developed on the open source OsiriX platform by manually defining endocardial and epicardial ventricular borders. Longitudinal and radial myocardial velocities were calculated from the RV free wall and the LV lateral wall.

## Results

**Figure 1:**
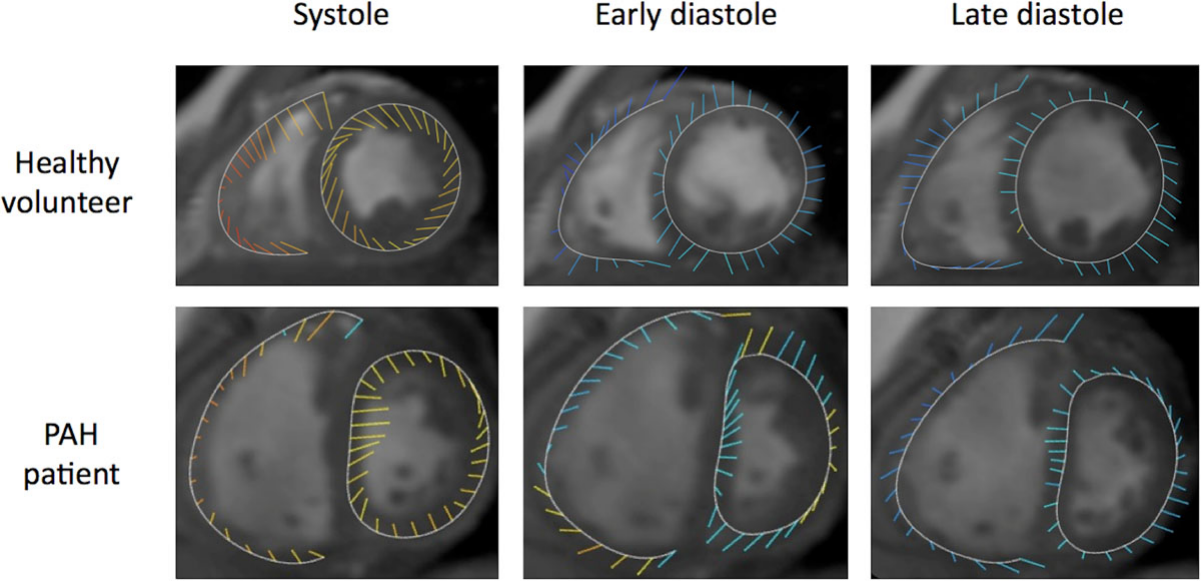
Myocardial motion by tissue phase mapping. The vectors represent myocardial velocity, with magnitude indicated by the vector length and longitudinal (through plane) direction indicated by the vector color. Yellow denotes longitudinal contraction, and blue denotes longitudinal relaxation. Note the reduced magnitude of right ventricular systolic radial velocities in PAH compared with the healthy volunteer data, and prolonged right ventricular radial contraction in early diastole associated with septal shift.

The self-gated sequence was successful in all patients with excellent image quality. Visualization of the motion vectors demonstrated obvious abnormalities in septal motion (Figure 1). Quantification revealed lower peak radial s-wave velocity in PAH compared to controls (1.5 cm/s ± 1.3 versus 2.3 ± 1.6, p < 0.02), as well as lower radial e-wave (1.2 ± 1.8 vs 2.4 ± 2.5, p < 0.03) and longitudinal e-wave (2.8 ± 3.5 vs 5.0 ± 3.2, p < 0.004) velocities. In addition, longitudinal a-wave velocity was higher in PAH compared to controls (6.4 ± 6.3 vs 4.1 ± 3.7, p < 0.03).

**Figure 2:**
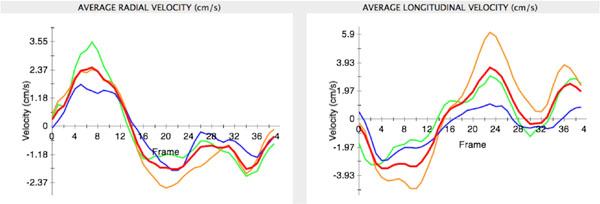
Radial and longitudinal velocity data from a healthy volunteer at the mid-cavity level of the right ventricle. The graphs display RV free wall (orange), anteroseptal (green), inferoseptal (blue) and average myocardial velocities, with clearly delineated systolic (s), early (e) and late (a) diastolic waves.

In patients with PAH, RV contraction was also significantly prolonged compared to LV contraction, both radially (46% ± 18 vs 42% ± 14 of cardiac cycle, p = 0.016) and longitudinally (39% ± 11 vs 33% ± 18, p = 0.013). There was no difference between RV and LV contraction times in normal volunteers (p > 0.35). Importantly, the prolongation of RV contraction significantly correlated with the degree of septal curvature (r = -0.54, p = 0.022).

## Conclusions

We have shown that it is possible to comprehensively assess RV myocardial motion using a novel free-breathing, golden-angle spiral TPM sequence. Using this technique we have shown in PAH that s-wave and e-wave velocities are reduced while a-wave velocities are increased, consistent with reduced RV systolic and diastolic function. Furthermore, we have shown that RV contraction time is prolonged in PAH and this correlated with septal curvature, implying a causal relationship. Thus, we believe that our novel TPM sequence is more fully able to assess ventricular dysfunction in PAH and may be an important tool in diagnosing PAH and monitoring response to therapy.

## Funding

Drs. Daniel Knight and Vivek Muthurangu are funded by the British Heart Foundation (BHF).

